# Vaginal Hysterotomy

**Published:** 1849-03

**Authors:** 


*Vaginal Hysterotomy.* By John H. Griffin, M. D., of Salem, Roanoke County, Virginia.

I was requested on Wednesday, May 10th, 1848, to visit Mrs. W., of Montgomery County, a young married lady of good constitution, in labour with her first child; but owing to existing professional engagements, was unable to go so far from home (twenty-six miles) on that day; being again sent for, however, that night, I reached my patient on the afternoon of the next day.

I found Drs. Eady, Kent and Jackson in attendance. Labour had commenced at midnight on the previous Monday, and up to that time, although the pain had been strong, no appearance of the "os tincæ" had been detected. After repeated careful examination, I was fully satisfied that the mouth of the uterus was altogether wanting. The external parts were rigid, and extremely sensitive to the touch; but within, what I supposed to constitute the neck of the uterus, was found soft, and spread out into a thin smooth membrane, through which the head of the child could be distinctly felt, and, between the two, during the existence of a pain, the bag-like projection of the distended membranes. But, after the most diligent search, no opening could be found, and nothing to mark the place where it should have been, except a slight degree of roughness, and apparent depression, at a spot not larger than a squirrel shot.

All the gentlemen present, having satisfied themselves of the condition of the patient, concurred in the opinion that the operation of vaginal hysterotomy should be no longer delayed, and requested that I would perform it.

After having the woman properly placed, I carefully sought for the rough depression above referred to, with the hope that I might be enabled to introduce a probe pointed bistoury, and thus effect the necessary division of the neck of the uterus, from a spot clearly indicating where the opening should have been. But in this I was disappointed, not being able to detect any opening, and was at length forced to substitute the sharp pointed for the blunt bistoury:—both of which had been previously prepared by wrapping them to within a short distance of their points. This was carried down to the same spot, between the fingers, and a free bi-lateral section made, which was afterwards somewhat enlarged, the probe-pointed bistoury being substituted for the sharp. No hemorrhage, worth mentioning, occurred, and, the section being made during the existence of a pain, the operation was performed without the knowledge of the patient.

The pains were now strong and frequent, and, although the presentation was the 6th of Baudelocque, and from the excessive rigidity and tenderness, it was impossible to induce the patient to submit to the entire introduction of the hand, in order to change it to one more favorable, yet it was thought best to delay any further interference, for a short time, with the hope that the natural efforts of the uterus would be adequate to the expulsion of the head. Disappointed in this, the mouth of the uterus being fully dilated, the application of forceps was proposed, and I proceeded to introduce the blades, amidst loud complaints of suffering. Some difficulty was experienced in locking them, and the patient, worn out by long suffering, with that impatience which might have been expected sooner to manifest itself, insisted that the instruments should be transferred to Dr. J. The blades were therefore reluctantly withdrawn, and handed over to that gentleman, who, after some effort, not succeeding in introducing them, again offered me his place at the bed-side, with the hope that my second effort would prove more fortunate than the first; but, as I was about introducing the second blade, the patient positively refused to submit to the operation, and her husband, also a physician, alarmed lest further delay should endanger the safety of his wife, now insisted that the crotchet should be immediately resorted to. My remonstrances were of no avail, and the head was extracted, mainly by the efforts of Dr. Eady, after *seventy two hours* of suffering, to the great relief of the mother, and delight of the friends present; but mingled, in my own case, with regret for the loss of the child, which, if alive at the time of the operation, ought, I think, to have been saved. The mother fortunately recovered, without any symptoms, so far as I have been able to learn, worthy of particular remark. The catamenia, which had been regular previous to marriage, returned in a few weeks.

I am informed by Dr. W. that, during the early stage of pregnancy, Mrs. W. complained of uneasiness and pain within the pelvis, which, at the time, was referred to the bladder; but which the occurrence above detailed renders probable was, in fact, inflammation of the neck of the uterus, by which the *os* was entirely obliterated; no opportunity has been presented for ascertaining its present condition.

This, according to Professor Bedford, of New York city, is the third time this operation has been performed in America.—*Am. Jour. Med. Sciences.*

See Buffalo Med. Jour., vol. II, for case by Prof. White; and vol. III, for case by Dr. Scott, of Buffalo. ED. BUFF. MED. JOUR.

